# A Systematic Mapping Approach of 16q12.2/*FTO* and BMI in More Than 20,000 African Americans Narrows in on the Underlying Functional Variation: Results from the Population Architecture using Genomics and Epidemiology (PAGE) Study

**DOI:** 10.1371/journal.pgen.1003171

**Published:** 2013-01-17

**Authors:** Ulrike Peters, Kari E. North, Praveen Sethupathy, Steve Buyske, Jeff Haessler, Shuo Jiao, Megan D. Fesinmeyer, Rebecca D. Jackson, Lew H. Kuller, Aleksandar Rajkovic, Unhee Lim, Iona Cheng, Fred Schumacher, Lynne Wilkens, Rongling Li, Keri Monda, Georg Ehret, Khanh-Dung H. Nguyen, Richard Cooper, Cora E. Lewis, Mark Leppert, Marguerite R. Irvin, C. Charles Gu, Denise Houston, Petra Buzkova, Marylyn Ritchie, Tara C. Matise, Loic Le Marchand, Lucia A. Hindorff, Dana C. Crawford, Christopher A. Haiman, Charles Kooperberg

**Affiliations:** 1 Division of Public Health Sciences, Fred Hutchinson Cancer Research Center, Seattle, Washington, United States of America; 2 Carolina Center for Genome Sciences, University of North Carolina at Chapel Hill, Chapel Hill, North Carolina, United States of America; 3 Department of Epidemiology, School of Public Health, University of North Carolina at Chapel Hill, Chapel Hill, North Carolina, United States of America; 4 Department of Genetics, School of Medicine, University of North Carolina at Chapel Hill, Chapel Hill, North Carolina, United States of America; 5 Department of Genetics, Rutgers University, Piscataway, New Jersey, United States of America; 6 Department of Statistics and Biostatistics, Rutgers University, Piscataway, New Jersey, United States of America; 7 Department of Internal Medicine, Ohio State Medical Center, Columbus, Ohio, United States of America; 8 Department of Epidemiology, Graduate School of Public Health, University of Pittsburgh, Pittsburgh, Pennsylvania, United States of America; 9 Department of Obstetrics and Gynecology, University of Pittsburgh, Pittsburgh, Pennsylvania, United States of America; 10 Epidemiology Program, University of Hawaii Cancer Center, Honolulu, Hawaii, United States of America; 11 Department of Preventive Medicine, Keck School of Medicine and Norris Comprehensive Cancer Center, University of Southern California, Los Angeles, California, United States of America; 12 Office of Population Genomics, National Human Genome Research Institute, National Institutes of Health, Bethesda, Maryland, United States of America; 13 Center for Complex Disease Genomics, McKusick-Nathans Institute of Genetic Medicine, Johns Hopkins University School of Medicine, Baltimore, Maryland, United States of America; 14 Division of Cardiology, Geneva University Hospital, Geneva, Switzerland; 15 Preventive Medicine and Epidemiology, Loyola University, Chicago, Illinois, United States of America; 16 Department of Medicine, University of Alabama, Birmingham, Alabama, United States of America; 17 Department of Human Genetics, University of Utah, Salt Lake City, Utah, United States of America; 18 Department of Biostatistics, University of Alabama, Birmingham, Alabama, United States of America; 19 Department of Biostatistics, Washington University, St. Louis, Missouri, United States of America; 20 Wake Forest University School of Medicine, Winston-Salem, North Carolina, United States of America; 21 Department of Biostatistics, University of Washington, Seattle, Washington, United States of America; 22 Pennsylvania State University, State College, Pennsylvania, United States of America; 23 Vanderbilt Epidemiology Center, Vanderbilt University Medical Center, Nashville, Tennessee, United States of America; 24 Center for Human Genetics Research, Vanderbilt University Medical Center, Nashville, Tennessee, United States of America; University of Oxford, United Kingdom

## Abstract

Genetic variants in intron 1 of the fat mass– and obesity-associated (*FTO*) gene have been consistently associated with body mass index (BMI) in Europeans. However, follow-up studies in African Americans (AA) have shown no support for some of the most consistently BMI–associated *FTO* index single nucleotide polymorphisms (SNPs). This is most likely explained by different race-specific linkage disequilibrium (LD) patterns and lower correlation overall in AA, which provides the opportunity to fine-map this region and narrow in on the functional variant. To comprehensively explore the 16q12.2/*FTO* locus and to search for second independent signals in the broader region, we fine-mapped a 646–kb region, encompassing the large *FTO* gene and the flanking gene *RPGRIP1L* by investigating a total of 3,756 variants (1,529 genotyped and 2,227 imputed variants) in 20,488 AAs across five studies. We observed associations between BMI and variants in the known *FTO* intron 1 locus: the SNP with the most significant p-value, rs56137030 (8.3×10^−6^) had not been highlighted in previous studies. While rs56137030was correlated at r^2^>0.5 with 103 SNPs in Europeans (including the GWAS index SNPs), this number was reduced to 28 SNPs in AA. Among rs56137030 and the 28 correlated SNPs, six were located within candidate intronic regulatory elements, including rs1421085, for which we predicted allele-specific binding affinity for the transcription factor *CUX1*, which has recently been implicated in the regulation of *FTO*. We did not find strong evidence for a second independent signal in the broader region. In summary, this large fine-mapping study in AA has substantially reduced the number of common alleles that are likely to be functional candidates of the known *FTO* locus. Importantly our study demonstrated that comprehensive fine-mapping in AA provides a powerful approach to narrow in on the functional candidate(s) underlying the initial GWAS findings in European populations.

## Author Summary

Genetic variants within the fat mass– and obesity-associated (*FTO*) gene are associated with increased risk of obesity. To better understand which specific genetic variant(s) in this genetic region is associated with obesity risk, we attempt to genotype or impute all known genetic variants in the region and test for association with body mass index as a measurement of obesity in over 20,000 African Americans. We identified 29 potential candidate variants, of which one variant (rs1421085) is a particularly interesting candidate for future functional follow-up studies. Our example shows the powerful approach of studying a large African American population, substantially reducing the number of possible functional variants compared with European descent populations.

## Introduction

The association between variants in the fat mass and obesity associated (*FTO*) gene on chromosome 16q12.2 and body mass index (BMI) is well-established in populations of European descent. Genome-wide association studies (GWAS) and subsequent replication studies have identified several strongly correlated single nucleotide polymorphisms (SNPs) located in intron 1 of *FTO* associated with increased BMI and increased risk of obesity [1]–[13]. With an observed effect size of 0.35 kg/m^2^ (0.1 z-score units of BMI) per risk allele, the *FTO* locus has a substantially stronger effect on BMI than any other identified common locus [14]. While this impact on BMI may seem small, it has a potential public health bearing, as even a 1 unit increase in BMI results in an estimated 8% increase in coronary heart disease [15], and excess weight in midlife is associated with increased mortality [16]. Thus, a seemingly small increase in BMI can have a marked impact, particularly in countries with an increasing burden of excess weight, such as the US, where an estimated 68% of adults were overweight or obese in 2008 [17].

Studies in non-European populations have had varied success in replicating the findings at the *FTO* locus. While several studies showed an association between *FTO* SNPs and obesity-related phenotypes in Hispanic [5], [6], [18], [19] and Asian populations [20]–[26], studies of African or African American (AA) subjects showed limited support for some of the most consistent *FTO* GWAS findings initially identified in subjects of European descent [2], [5], [6], [14], [18], [19], [27]–[32]. This lack of generalization in AA may be attributable to the lower levels of linkage disequilibrium (LD) with the underlying functional variant(s) at the 16q12.2/*FTO* locus, as compared with European Americans (EAs). SNPs discovered in GWAS (i.e. “index SNPs”) are often not the functional variants; however, they do tag genomic regions harboring strongly correlated variants, one or more of which are the potentially functional variant(s). Because different ancestral populations differ in their LD patterns, an index SNP discovered in one ancestral group (e.g. EA) may or may not be strongly correlated with the functional variant(s) in a different ancestral group (e.g. AA). Thus, the index SNP may not show evidence for replication in AAs; however, other SNPs in the region may be in high LD with the functional variant(s), and, hence, measuring these SNPs may further characterize associations within the genomic region. Therefore, a full exploration of potential replication/generalizability of GWAS findings in other ancestral groups requires investigating not only at the index SNP(s), but also examining, if possible, all variants of the region tagged by the index SNP(s). African populations are particularly suited for these studies because the LD pattern between SNPs tends to be substantially weaker than in other ancestral groups, as has been demonstrated for the *FTO* gene [30]. This process can reduce the number of potential functional variants for follow-up molecular investigation [33]. Given that functional studies can be labor- and cost-intensive, narrowing the associated region is an important step toward elucidating the underlying molecular mechanism. While molecular evaluation of the 16q12.2/*FTO* locus provides some promising leads [34], the putative functional variant(s) in this locus remain under investigation, and fine-mapping studies have been limited with respect to the number of tested variants, sample sizes and inclusion of non-EA populations. Fine-mapping studies, particularly when conducted within a broader region, may also identify additional independent signal(s) implicating multiple functional variants in the region. To better understand the relationship of genetic variation at this locus and BMI in AAs, we comprehensively assessed the association of BMI with variants in the 16q12.2/*FTO* region and flanking gene *RPGRIP1L* in over 20,000 AA samples from the Population Architecture using Genetics and Epidemiology (PAGE) consortium. We used the Metabochip as genotyping platform [35], which included all suitable SNPs discovered in the 1000 Genomes Project Pilot 1 for the 16q12.2/*FTO* region and led to successfully genotyping of over 1,500 SNPs. This together with imputation into the updated 1000 Genomes Project allowed us to densely fine-mapping this region. In addition, we performed a detailed bioinformatic analysis to propose candidate polymorphisms for follow-up functional evaluation.

## Results

The age of the 20,488 participants ranged from 20 to 85 years with an average age of 58.5 years across the cohorts (Table 1). The fraction of men included in each cohort varied from 0% to 43%. In all studies that included both genders, men had on average a lower BMI than the women. Participants in the Hypertensive Genetic Epidemiology Network (HyperGEN) had the highest BMI while participants in the Multiethnic Cohort (MEC) had the lowest BMI. The obesity rate (defined as BMI≥30 kg/m^2^) across the studies was 46% ranging from 31% to 58%.

**Table 1:** Distribution of study characteristics by study and overall.

Characteristics	ARIC (n = 3,297)		GenNet (n = 517)		HyperGen (n = 1,171)		MEC (n = 3,865)	
	Male	Female	Male	Female	Male	Female	Male	Female
**Sex**	1,224 (37)	2,073 (63)	221(43)	296 (57)	391 (33)	780 (67)	1,037 (27)	2,828 (73)
**Age**								
**Mean (+/−SD)**	53.8 (6.0)	53.4 (5.7)	38.2(7.1)	38.4(8.0)	47.6 (12.5)	48.0 (12.4)	61.9 (8.0)	58.6 (8.8)
**Range (max-min)**	44–66	44–66	22–64	20–62	21–85	20–81	45–76	45–77
**Height (m)**								
**Mean (+/−SD)**	176.2 (6.7)	163.2 (6.2)	176.9(7.2)	164.3(6.4)	175.8 (7.0)	162.4(6.2)	175.0 (6.5)	161.3 (6.4)
**Range (max-min)**	153–197	125–188	155–196	138–183	155–198	145–183	153–198	133–198
**Weight (kg)**								
**Mean (+/−SD)**	86.7 (16.5)	82.4 (17.8)	86.8(22.8)	88.8(25.2)	93.0(21.9)	89.3(22.0)	86.3 (14.3)	77.6 (16.6)
**Range (max-min)**	50.9–165.9	44.5–177.3	54.9–204.3	49.5–175.2	54.9–181.4	45.4–210.0	49.0–179.3	43.6–181.6
**BMI (kg/m2)**								
**Mean (+/−SD)**	27.9 (4.9)	31.0 (6.5)	27.4(6.9)	32.9(9.2)	30.0(6.3)	33.7(7.6)	27.3 (4.2)	28.9 (6.0)
**Range (max-min)**	18.6–54.4	18.5–65.9	18.8–62.8	18.9–63.2	18.6–52.4	19.4–62.7	18.6–60.2	18.6–64.7
**Obesity (BMI> = 30), n (%)**	360 (29)	1010 (49)	63 (29)	161 (54)	181(46)	499(64)	212 (20)	990 (35)

Characteristics	WHI genotyped	WHI imputed	All (n = 20,488)		
	Female (n = 5,312)	Female (n = 6,326)	Male	Female	Both
**Sex**	5,312 (100)	6,326 (100)	2,873 (14)	17,615 (86)	20,488 (100)
**Age**					
**Mean (+/−SD)**	61.2(7.2)	61.8 (7.1)	54.7 (8.0)	59.1 (7.6)	58.5 (7.7)
**Range (max-min)**	50–79	50–79	22–85	20–81	20–85
**Height (m)**					
**Mean (+/−SD)**	162.6 (6.3)	162.4 (6.4)	175.7 (6.7)	162.4 (6.3)	164.3 (6.4)
**Range (max-min)**	100–192	117–194	153–198	100–198	100–198
**Weight (kg)**					
**Mean (+/−SD)**	83.3 (18.4)	81.9 (17.7)	87.4 (17.2)	82.1 (18.1)	82.9 (18.0)
**Range (max-min)**	45.0–187.0	39–181	49.0–204.3	39–210	39.0–210
**BMI (kg/m2)**					
**Mean (+/−SD)**	31.5 (6.8)	31.0 (6.4)	27.9 (5.1)	31.0 (6.6)	30.5 (6.4)
**Range (max-min)**	18.6–68.3	18.6–69.2	18.6–62.8	18.5–69.2	18.5–69.2
**Obesity (BMI> = 30), n (%)**	2806 (53)	3185 (50)	816 (28)	8651 (49)	9467 (46)

**Figure 1:**
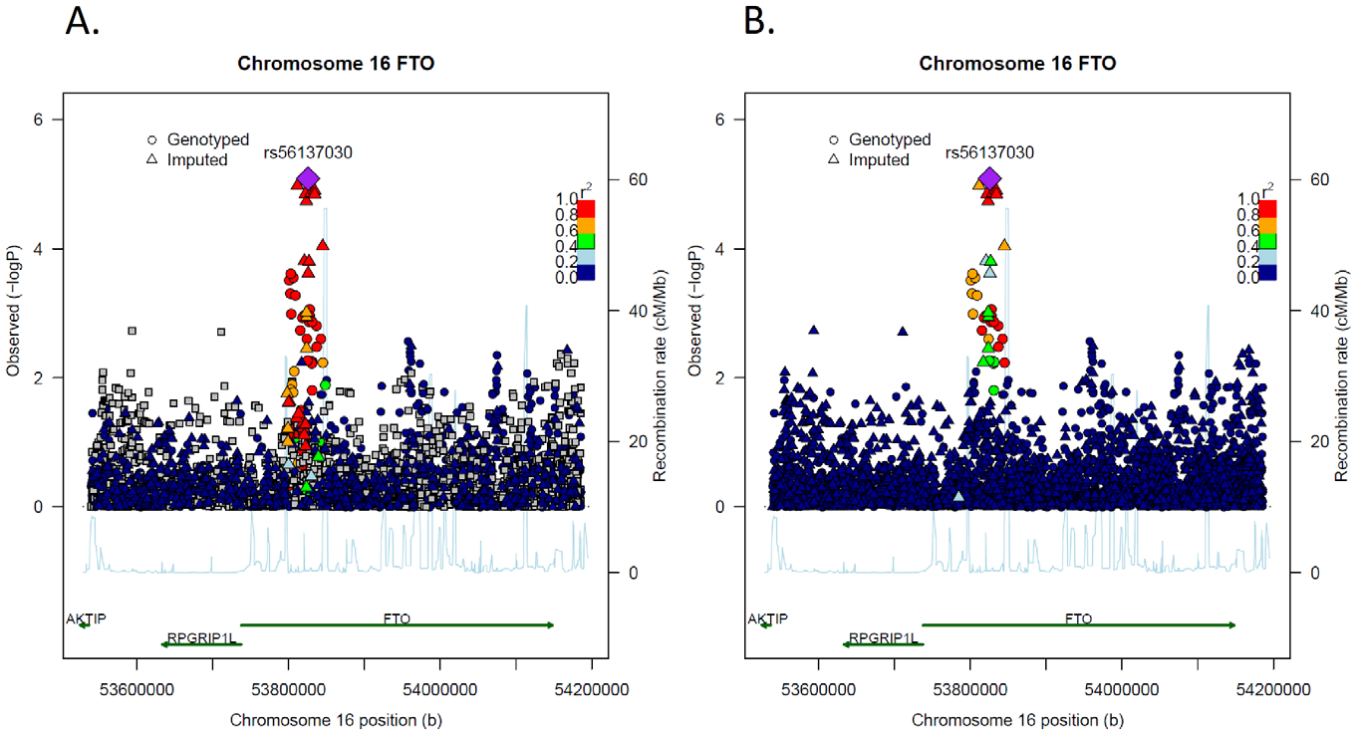
Association results (p-values) and correlation structure for all SNPs in the 16q12.2/*FTO* region and lnBMI among African Americans using rs56137030 to calculate correlation among SNPs (LocusZoom plots). The top half of each figure has physical position along the x axis, and the −log_10_ of the meta-analysis p-value on the y-axis. Each dot on the plot represents the p-value of the association for one SNP with lnBMI across all studies. The most significant SNP (rs56137030) is marked as a purple diamond. The color scheme represents the pairwise correlation (r^2^) for the SNPs across the 16q12.2/*FTO* region with the most significant SNP (rs56137030). Gray squares indicate that correlation was missing for this p-value because the variant was monomorphic in EA. The bottom half of the figure shows the position of the genes across the region. A and B show the same region and results. The only difference between A and B is that in A correlation with the most significant SNP (rs56137030) was calculated based on EAs, specifically based on data from 65 European Americans (Utah residents with Northern and Western European ancestry from the CEPH collection, CEU) sequenced as part of the 1000 Genomes Project and B correlation was based on 61 African Americans from the South-west (ASW) and sequenced as part of the 1000 Genomes Project.

The targeted 16q12.2/*FTO* fine-mapping region spans 646 kb from 53,539,509 to 54,185,773 (build 37) on the long arm of chromosome 16 (16q12.2), including the large *FTO* gene (411 kb) as well as 198 kb downstream of *FTO*, which includes the *RPGRIP1L* gene and 37 kb upstream of *FTO* (Figure 1). On average, we successfully genotyped or imputed one SNP per 172 bp ( = 646,264 bp/3,756 SNPs). The allele frequency distribution of the 3,756 SNPs in this region is shown in Table 2. In contrast to GWAS platforms, a large fraction of SNPs have allele frequencies <5% (52.5%), including 16.7% with allele frequency <1%. Twenty-one SNPs are within the exons of *FTO* and *RPGRIP1L*, of which 7 are synonymous and 14 are missense (2 synonymous and 4 missense are located in *FTO*).

**Table 2:** Distribution of allele frequency of SNPs in the *FTO* fine-mapping region.

SNP	n	%
**Allele frequency** a		
0.14%–1%	627	16.7%
>1%–5%	1346	35.8%
>5%–10%	431	11.5%
>10%–25%	679	18.1%
>25%	673	17.9%
Total	3756	100.0%
**Exonic SNPs** b		
SNPs in *RPGRIP1L* exons	15	0.4%
synonymous SNPs in *RPGRIP1L*	3	0.1%
missense SNPs in *RPGRIP1L*	12	0.3%
SNPs in *FTO* exons	6	0.2%
synonymous SNPs in *FTO*	4	0.1%
missense SNPs in *FTO*	2	0.1%

**a**Based o allele frequency for all AA participants in this study.

**b**Based on data from the Exome Variation Server (http://snp.gs.washington.edu/EVS/ accessed August 2012).

The most significant SNPs in the 16q12.2/*FTO* fine-mapping region was rs56137030 (p-value 8.3×10^−6^), which showed no evidence for heterogeneity (p = 0.13; Table 3). Each A allele of this variant (allele frequency = 0.12) increased BMI by 1.35% (95% confidence interval (0.76%–1.95%). Table 3 also displays results for the three next most significant SNPs showing similar significant associations (p-values 1.4×10^−5^ to 1.1×10^−5^) and Table S1 shows results separately for each study. All three SNPs were correlated with rs56137030 (r^2^≥0.73 in AA and r^2^≥0.91 in EA; Table S2). rs56137030 and the three next most significant SNPs are located in intron 1 of the *FTO* gene, approximately in the middle of the region (Figure 1). The nine GWAS index SNPs previous highlighted in EA studies are also located in the same intron 1 *FTO* region and results for these nine GWAS index SNPs are provided in Table 3 showing p-values<0.05 for five out of the nine index SNPs (0.03 to 3.0×10^−4^).

**Table 3:** Association between SNPs in the *FTO* region and BMI for all studies combined.

SNP		Alleles^b^		CAF^c^	% change in BMI per coding allele		nom.p	Rsq^d^	p.het
rs#	Position^a^	Coding^b^	Baseline^b^		Beta estimate	95%CI			
**Five most significant variants** (all in the *FTO* intron 1 region)									
rs56137030	53825905	A	G	0.12	1.35	(0.76,1.95)	8.3E-06	0.98	1.3E-01
rs62033400	53811788	G	A	0.12	1.35	(0.76,1.95)	1.1E-05	0.99	1.3E-01
rs7188250	53834607	C	T	0.12	1.34	(0.75,1.94)	1.3E-05	0.97	8.3E-02
rs62033413	53830055	G	C	0.12	1.33	(0.74,1.93)	1.4E-05	0.98	4.5E-02
**Index SNPs of GWAS** (variants highlighted in previous studies of EA; all in *FTO* intron 1 region)									
rs9939609	53820527	T	A	0.52	0.04	(−0.33,0.41)	8.2E-01	1.00	5.5E-01
rs8050136	53816275	A	C	0.44	0.42	(0.03,0.82)	3.2E-02	1.00	4.9E-02
rs1421085	53800954	G	A	0.12	1.11	(0.49,1.72)	3.0E-04	1.00	2.6E-01
rs17817449	53813367	C	A	0.39	0.37	(−0.02,0.77)	5.9E-02	1.00	5.6E-02
rs1121980	53809247	A	G	0.47	0.35	(−0.02,0.73)	7.2E-02	1.00	2.4E-01
rs1558902	53803574	T	A	0.12	1.13	(0.46,1.80)	1.0E-03	1.00	2.4E-01
rs6499640	53769677	A	G	0.65	0.05	(−0.34,0.44)	8.1E-01	1.00	1.6E-01
rs9930506	53830465	G	A	0.22	0.66	(0.19,1.14)	5.5E-03	1.00	1.6E-01
rs9941349	53825488	A	G	0.12	0.69	(0.20,1.19)	5.4E-03	1.00	5.7E-01

**a**SNPposition based on build 37.

**b**Coding = coding allele, Base = baseline allele (risk estimates provide the log additive effect per copy of the coding allele).

**c**CAF = coding allele frequency.

**d**Rsq = measurement of imputation accuracy, ranging from 0 (low) to 1 (high).

The most significant variant, rs56137030, was correlated at r^2^>0.5 with 103 SNPs in Europeans (Figure 1a), including eight of the nine GWAS index SNPs (Table S2). In contrast in AAs rs56137030 was correlated with only 28 SNPs at r^2^>0.5 (Table S2, Figure 1b). All 28 variants correlated with rs56137030 in AA showed some evidence of association with BMI (p-values 0.0057 to 1.1×10^−5^) and no or limited evidence of heterogeneity (all p for heterogeneity >0.04). To investigate if any of these correlated SNPs were associated with BMI independently from rs56137030 we adjusted each SNP for rs56137030 (including rs56137030 and a second SNP simultaneously in one model). None of these variants remained significant at p<0.05 (Table S2). As expected, the p-values of rs56137030 were also less significant in these conditional analyses, particularly for SNPs highly correlated in AA, demonstrating that these findings are not independent. All 28 SNPs correlated with rs56137030 (r^2^>0.5) in AA are located between 53,800,954 and 53,845,487, spanning a 44.5 kb region about 104.8 kb downstream of the exon 1 boundary, and ending about 1.4 Kb after exon 2 ends [no variant in exon 2 was genotyped or imputed and, to our knowledge, only 3 variants (rs116753298, rs149393601, and chr16:53844100) all with allele frequency <0.1% have been reported in *FTO* exon 2 so far [36], [37]].

To predict the molecular mechanisms underlying the genetic association signals, and to identify candidate variants for functional follow-up, rs56137030 and variants in LD (r^2^>0.5; n = 28) were assessed for overlap with eleven different genome-wide functional annotation datasets (Table S3, Material and Methods). Among these 29 variants, six (rs11642015, rs17817497, rs3751812, rs17817964, rs62033408, and rs1421085) were located within candidate intronic regulatory elements, including two (rs3751812 and rs1421085) that were within highly sequence-conserved elements among vertebrates, and two (rs11642015 and rs1421085) that were predicted to have allele-specific binding affinities for different transcription factors. Specifically, we predicted that only the T allele at rs11642015 binds Paired box protein 5 (*PAX5*) and that the C allele at rs1421085 has a substantially reduced binding affinity for Cut-like homeobox 1 (*CUX1*; Figure S1).

Outside the known *FTO* intron 1 region, we observed no strong evidence for a second independent signal: When we adjusted each SNP for rs56137030 the most significant SNPs outside of the *FTO* intron 1 region was a SNP located at position 53710931 (no rs number reported) intronic of the neighbor gene *RPGRIP1L* (conditional p-value 7.7×10^−4^) followed by rs8051873 in intron 8 of *FTO* (conditional p-value 0.0011).

Table S4 shows results for variants highlighted in previous AA studies. Of these nine SNPs only two SNPs had a p-values<0.05 (rs3751812, p value 0.0012).

## Discussion

In this large study of over 20,000 AAs we densely fine mapped the entire *FTO* gene and adjacent *RPGRIP1L* gene, spanning a total region of almost 650 kb. We observed significant associations for variants in the known locus in intron 1 of *FTO*. Due to reduced correlation in AA compared with EA with the most significant SNPs, we were able to substantially reduce the number of functional candidates. Six SNPs were located within candidate intronic regulatory elements, including rs1421085, for which we predicted allele-specific binding affinity for the transcription factor *CUX1*. Because we did not focus solely on the known *FTO* intron 1 region, we were able to comprehensively investigate the region; however, this approach revealed no evidence for a second independent signal in AA.

This is one of the first studies of the Metabochip in an ancestral group that is particularly well suited for fine-mapping GWAS loci, due to its distinct linkage disequilibrium (LD) patterns and lower LD overall. Our example clearly shows the powerful approach of studying a large AA population, substantially reducing the number of possible functional variants compared with European descent populations. While very large number of Europeans and EAs are genotyped on the Metabochip, the number of Minority populations genotyped on this chip is substantially smaller; however, this is the focus of the PAGE Study. As many papers using the Metabochip in European populations will be published over the next years, our results show the important contribution that Minority populations and, in particularly AA, will have for systematic mapping of GWAS loci.

The importance of the 16q12.2/*FTO* locus for obesity-related traits was identified in genome-wide scans of Europeans. These scans highlighted several variants within the *FTO* intron 1, all of which, except for rs6499640, are in high LD with each other in EA [1]–[7]. Consistently these studies showed an increase in BMI (∼1.1% to 1.3% per risk allele; [7]). However, AA studies showed very limited or no evidence for an association with rs9939609 [2],[5],[6],[14],[18],[28]–[31], rs1121980 [18], [19], [28], [30], [31], rs17817449 [18], [19], [28]–[31], or the previously reported functional variant rs8050136 [2], [6], [19], [27]–[29], [31], [32]. We observed nominal evidence for association with BMI for rs17817449 and rs8050136, but results were not among the most significant associations. rs1421085 was significantly associated in our study (p = 3×10^−4^;Table S2) and was also found to be associated in the study from Nock et al. [29] (n = 469, p-value = 7×10^−4^), Hassanein et al. [31] (n = 4,217, p-value = 3×10^−4^), and Hester et al. [32] (n = 4,992, p-value = 0.07) but not with four smaller AA studies (≤1000 AA subjects) [5], [18], [28], [38]. Consistent with our finding Hassanein et al. [31] also observed a significant association with rs9941349 and rs1558902 (n = 9,881, p-value = 4×10^−6^ and n = 4,217, p-value = 2×10^−5^, respectively), these SNPs have not been genotyped in other AA studies. However, to put these findings into context with other variants in this region a comprehensive evaluation of all variants is needed.

To conduct a more comprehensive evaluation of the *FTO* locus, some AA studies extended the SNPs list from the EA index SNPs described above (Table S4). Grant et al. [27] analyzed eleven *FTO* SNPs genotyped as part of their GWAS in about 2,000 AA children and only rs3751812 showed a marginally significant association with BMI (p-value = 0.02). Wing et al. [18], [19] genotyped up to 27 SNPs in the intron 1 of *FTO* in a cohort study and family study including 288 and 604 AA, respectively, and observed an association of BMI with rs1108102 (p-value = 5×10^−4^; [18]); however, this finding was not confirmed in their cohort study or in our larger meta-analysis (Table S4). A fine mapping study of 47 tagging SNPs in 497 AA children [28] identified an association of rs8057044 (p-value = 5×10^−4^) with BMI, which was not replicated by the current study. In two fine-mapping studies [5], [14], no *FTO*-BMI associations were noted, possibly because the majority of subjects were lean (∼75% had a BMI between 18–25) in one study [14], or because the sample size was small in both studies (about 1,100). Hassanein et al. [31] genotyped 34 tagging SNPs in the *FTO* intron 1 region in 4,217 AA and followed up findings from two variants (rs3751812, p-value = 4×10^−4^ and rs9941349, p-value = 6×10^−5^) in four additional studies (n = 5,664), adding some support (p-values ranging from 0.016 to 0.64) which resulted in overall p-values of 2.6×10^−6^ and 3.6×10^−6^, respectively. The authors concluded that this finding reduced the potential functional variants to those correlated with these two variants. Both variants (rs3751812 and rs9941349) were also associated in our meta-analysis (p-value 0.001 and 0.005, respectively; Table S4), although they were not among the most significant findings. In summary, except for Hassanein et al. [31], studies in AA were relatively small and showed mixed results that were mainly not replicated in our study. As the number of variants genotyped in any AA study was limited (10 to 50 SNPs), we were able to investigate if genotyping or imputing additional variants in this region (in total 3,756) may even further reduce the list of possible functional variants and search for second independent signal(s).

In our analysis, rs56137030 was most significantly associated with BMI. In EA, rs56137030 has a similarly high allele frequecy as the previously reported GWAS index SNPs (A allele frequency = 0.42) and was highly correlated with the GWAS index SNPs (r^2^≥0.87), except for rs6499640 (r^2^ = 0.12), which is also not strongly correlated with any of the other GWAS index SNPs in EA or AA. In AA, the correlation between rs56137030 and GWAS index SNPs varied substantially (r^2^ = 0.001 to 0.73), demonstrating that studying AA can substantially reduce the bin of correlated SNPs defined by the index signal identified in EA. Specifically, the number of SNPs correlated with rs56137030 at r^2^>0.5 was 103 in EA, but only 28 in AA. Including these 28 SNPs together with rs56137030 in conditional analyses showed that the significance of each of the SNPs, as well as rs56137030, was substantially reduced, supporting the idea that any of the highly correlated SNPs is a potential functional candidate. rs56137030 and all 28 highly correlated SNPs are non-coding, suggesting that the functional variant is likely to have a *cis*-regulatory effect. Among these variants, six are located within the candidate intronic regulatory elements, two of which are highly conserved, and two that are predicted to confer allele-specific binding affinities for transcription factors. The variant rs1421085 is within a highly conserved element and may be particularly interesting, because the C allele has a substantially reduced binding affinity for *CUX1*, which has been previously implicated in the transcriptional regulation of *FTO* [34]. Accordingly, this variant is a compelling candidate for follow-up functional evaluation, though outside the scope of the present study.

We did not observe any evidence for a second independent signal within the broader 16q12.2/*FTO* region. This finding is consistent with the only other AA study that extended the fine-mapping approach beyond intron 1 to the entire *FTO* gene including 262 tagSNPs in 1,485 subjects [30]. However, even within our substantially larger study of over 20,000 AAs, power to identify second independent signals may still have been limited, particularly for less frequent variants or variants with weaker effects, given the increased burden of multiple comparisons that needs to be adjusted for when testing all SNPs across the entire region.

Several limitations warrant consideration to inform fine-mapping and functional characterization studies. To comprehensively evaluate the region we not only included directly genotyped SNPs, which included all SNPs known at the time of the chip development and suitable for genotyping SNPs but we also imputed to the most recent version of the 1000 Genomes Project. While this approach provides a rather complete list of variants imputed SNPs ted to be called with varying accuracy. To account for the imputation accuracy we used the dosage, which we showed results in unbiased estimates [39]. However, we also note that the overall p-value of a SNP is impacted by the imputation accuracy (lower imputation accuracy results in higher p-values). Accordingly, it is important for the interpretation of the results that not only the most significant SNPs will be considered as functional variants but also those correlated with the most significant SNPs as done in this paper. Second, for a part of the WHI samples directly genotyped SNPs were only available from a smaller subset of SNPs as genotyping in this subset was based on a GWAS platform and not the dense Metabochip. However, the imputation Rsq as a measurement of the accuracy was very high (Table S1). Third, despite the relative large samples size of over 20,000 AA the statistical significance of the finding is relative weak compared to previous studies in European descent populations for the *FTO* region. We note that the relative weak power of our study is not due to differences in the observed effect size, e.g. we observed a 1.35% change in BMI per allele for the most significant SNP while the replication stage of Willer et al. [7] observed a 1.25% change in BMI per allele of their most significant *FTO* SNP (rs9939609). However, the substantially lower allele frequency in AA compared with EA (12% vs. 41%) and the larger variance of BMI in AA populations (e.g. the standard deviation in our study was 6.4 kg/m^2^ compared with 4.2 kg/m^2^ in European populations [7] explains the reduce power. Fourth, our functional characterization is based on in silico analyses and requires experimental validation. Finally, the majority of study participants were female and it is unclear how a predominantly female population may have influenced the results.

To our knowledge, this is the largest and most comprehensive fine-mapping study conducted to date in AA. Our findings likely rules out that several of the EA index SNPs in intron 1 of *FTO* such as rs9939609, as well as a large fraction of SNPs correlated in EA but not in AA are the underlying functional variants. With rs56137030 and its correlated SNPs, our finding points us closer to the functional variant(s). Among these, rs1421085 is the most compelling candidate for follow-up functional evaluation. Importantly, our study demonstrated that comprehensive fine-mapping in AA provides a powerful approach to narrow in on the functional candidate(s) underlying the initial GWAS findings in EA.

## Materials and Methods

### Ethics statement

All studies were approved by Institutional Review Boards at their respective sites, and all participants provided informed consent.

### Study populations

PAGE involves several studies, described briefly below and in more detail in Text S1 as well as at the PAGE website (https://www.pagestudy.org) [40]. In brief, participants were recruited from Atherosclerosis Risk in Communities Study (ARIC), GenNet, Hypertension Genetic Epidemiology Network (HyperGEN), Multiethnic Cohort (MEC), and Women's Health Study (WHI). ARIC randomly selected and recruited 15,792 participants aged 45–64 at four U.S. communities [41]. GenNet and HyperGEN are two family-based studies designed to investigate the genetics of hypertension and related conditions [42]. The Multiethnic Cohort (MEC) is a population-based prospective cohort study of over 215,000 men and women in Hawaii and California aged 45–75 at baseline (1993–1996) and primarily of five ancestries [43]. The WHI encompasses four randomized clinical trials as well as a prospective cohort study of 161,808 post-menopausal women aged 50–79, recruited (1993–1998) and followed up at 40 centers across the US [44]. All studies collected self-identified racial/ethnic group via questionnaire. We selected all AA participants from ARIC, HyperGEN, and GenNet for genotyping. In MEC, a subset of AAs was selected based on availability of biomarker or as controls for nested case-control studies. WHI included all AAs who provided consent for DNA analysis. We excluded underweight (BMI<18.5 kg/m^2^) and extremely overweight (BMI>70 kg/m^2^) individuals with the assumption that these extremes could be attributable to data coding errors, an underlying illness or possibly to a familial syndrome and hence, a rare mutation. We also limited analysis to adults defined as age >20 years.

### Anthropometric measurements

For ARIC HyperGEN, GenNet and WHI, BMI was calculated from height and weight measured at time of study enrollment. In MEC, self-reported height and weight were used to calculate baseline BMI. A validation study within MEC has shown high validity of self-reported height and weight. Specifically this study showed that BMI was under-estimated based on self-reported compared to measured weight, but the difference was small (<1 BMI unit) and comparable to the findings from national surveys [45].

### SNP selection for genotyping

SNPs were included as part of the Metabochip, a 200 k Illumina customized iSelect array developed through the collaborative efforts of several consortia working on metabolic syndrome related diseases. Details on the design can be found elsewhere (http://www.sph.umich.edu/csg/kang/MetaboChip/). In brief, SNPs within the 16q12.2/*FTO* region were selected based on 1000 Genomes Pilot 1 and HapMap phase 2. The boundaries around each GWAS index SNP were determined by identifying all SNPs with r^2^≥0.5 with the index SNP, and then expanding the initial boundaries by 0.02 cM in either direction using the HapMap-based genetic map [46]. The total interval size of the 16q12.2/*FTO* region was 646 kb. All 1000 Genomes Pilot 1 SNPs obtained from Sanger Institute (August 12, 2009) and the Broad Institute (August 11, 2009) were considered as potential fine mapping SNPs, unless SNP allele frequency was <0.01 in all three HapMap samples (CEU, YRI and HBC/JPT). SNPs were excluded if (a) the Illumina design score was <0.5 or (b) there were SNPs within 15 bp in both directions of the SNP of interest with allele frequency of >0.02 among Europeans (CEU). SNPs annotated as nonsynonymous, essential splice site, or stop codon were included regardless of allele frequency, design score, or nearby SNPs in the primer.

### Genotyping and quality control

Samples were genotyped on the Metabochip at the Human Genetics Center of the University of Texas-Houston (ARIC, GenNet and HyperGEN), the University of Southern California Epigenome Center (MEC), and the Translational Genomics Research Institute (WHI). Each center also genotyped 90 HapMap YRI (Yoruba in Ibadan, Nigeria) samples to facilitate cross-study quality control (QC), as well as 2–3% study-specific blinded replicates to assess genotyping reproducibility. Genotypes were called separately for each study using GenomeStudio with the GenCall 2.0 algorithm. Samples were called using study-specific cluster definitions (based on samples with call rate >95%, ARIC, MEC, WHI) or cluster definitions provided by Illumina (GenNet, HyperGEN) and kept in the analysis if call rate was >95%. We excluded SNPs with GenTrain score <0.6 (ARIC, MEC, WHI) or <0.7 (GenNet, HyperGEN), cluster separation score <0.4, call rate <0.95, and Hardy-Weinberg Equilibrium p<1×10^−6^. We also excluded SNPs based on Mendelian errors in 30 YRI trios >1, replication errors >2 with discordant calls (when comparing across studies) in 90 YRI samples >3, and discordant calls for 90 YRI genotyped in PAGE versus HapMap database >3. In total, we successfully genotyped 1,694 out of 1,818 variants in the 16q12.2/*FTO* region. After excluding 165 SNPs that were monomorphic or had very low allele frequency (<0.01%) we included 1,529 variants in the analysis.

For ARIC, MEC and WHI we identified related persons using PLINK by estimating identical-by-descent (IBD) statistics for all pairs. When apparent first-degree relative pairs were identified, we excluded from each pair the member with the lower call rate. We excluded from further analysis samples with an inbreeding coefficient (F) above 0.15 (ARIC, MEC, WHI) [47]. We determined principal components of ancestry in each study separately using EIGENSOFT [48], [49] and excluded apparent ancestral outliers from further analysis as described elsewhere [50]. In total 240 subjects failed genotyping (ARIC = 27, GenNet = 9, HyperGEN = 26, MEC = 140, and WHI = 27). After further excluding subjects based on age and BMI (see above), a total of 14,162 subjects with Metabochip data were included (3,297 from ARIC, 517 from GenNet, 1,171 from HyperGEN, 3,865 from MEC, and 5,312 from WHI). In addition we included 6,326 WHI participants genotyped as part of the SNP Health Association Resource (SHARe) on the Affymetrix 6.0 platform. Details can be found elsewhere [51].

### Imputation to 1000 Genomes Project

To impute to the 1000 Genomes Project we used as the reference panel the haplotypes of the 1092 samples (all populations) from release version 2 of the 1000 Genomes Project Phase I (ftp://ftp-trace.ncbi.nih.gov/1000genomes/ftp/release/20110521) [52]. Combining reference data from all populations has been found to improve imputation accuracy of the low-frequency variants [53], and, hence, is recommended. We built the target panel by combining all genotype data in the *FTO* region from all studies. We used genotyped data from the Metabochip for all studies, except for WHI SNP Health Association Resource (SHARe, n = 6,326 samples) where we used genotype data from the Affymetrix 6.0 platform. The target panel was phased using Beagle [54]. We then performed a haplotype-to-haplotype imputation to estimate genotypes (as allele dosages) at 1000 Genomes Project variants. The phased target panel was imputed to the interval 53.5–54.2 Mb on chromosome 16 of the 1000 genomes reference panel using minimac [55]. To evaluate the quality of each imputed SNP we calculated Rsq. We excluded imputed SNPs with Rsq<0.9 for SNPs with allele frequency <0.5%, Rsq<0.8 for SNPs with allele frequency >0.5–1%, Rsq<0.7 for SNPs with allele frequency >1–3%, Rsq<0.6 for SNPs with allele frequency >3–5%, and Rsq<0.5 for SNPs with allele frequency >5%. Given the large reference panel this resulted in high imputation quality [51]. To calculate pairwise correlation between variants we used the 1000 Genomes Project data, specifically we used 61 African Americans from the South-west (ASW) and 65 European Americans (Utah residents with Northern and Western European ancestry from the CEPH collection, CEU).

### Statistical analysis

The association between each SNP and natural log-transformed BMI (lnBMI) was estimated using linear regression. SNP genotypes were coded assuming an additive genetic model (i.e., 0, 1, or 2 copies of the coded allele). All analyses were adjusted for age (continuous), sex, and study site (as applicable). All models (except WHI) included sex*age interaction terms to account for possible effect modification by sex. In addition, we adjusted for the top two principal components of ancestry. Family data from GenNet and HyperGEN was analyzed using mixed models (variance component models) to account for relatedness.

Results (β and SEs for lnBMI) were combined with fixed-effects meta-analysis weighting the effect size estimates (β-coefficients) by their estimated standard errors, using METAL [56]. We evaluated Q-statistic and I^2^ as a measure of heterogeneity [57], [58], to describe the presence or absence of excess variation between the PAGE cohorts. For ease of interpretation, we calculated the % change in BMI per copy of the effect allele based on the beta for the lnBMI. To graphically display the results, we used LocusZoom [59]. We tested for independence of findings by including the most significant variant and each of the other variant into the same model (i.e. we included 2 variants simultaneously in one model). We evaluated if SNPs are independent by investigating the p-value. For an independent SNP the p-value would remain low/similar after adjusted for the most significant SNP.

### Bioinformatics

We conducted a bioinformatic characterization for the most significant SNP and all SNPs correlated with the most sigificant SNP (r^2^>0.5). We implemented in-house Perl scripts to query bioinformatic databases, and assigned each of the 16 SNPs to one or more of the functional annotation datasets listed in Table S3. These datasets are not mutually exclusive. For example, a SNP can be located within both a candidate regulatory element (dataset #7) and a CTCF binding site (dataset #10). Because FTO is expressed and may have functional relevance in a wide array of tissues, we defined candidate *cis*-regulatory elements (dataset #7) as DNaseI hypersensitive sites (open chromatin loci) that are present in at least one human cell type. For SNPs that occur within predicted transcription factor binding sites (datasets #3 and #8), we computed transcription factor binding affinity for each SNP allele using the PWM-scan algorithm [60], as described previously [61].

## Supporting Information

Figure S1 Predicted CUX1 binding site at the rs1421085 locus.
(DOCX)

Table S1 Association between SNPs in the *FTO* region and BMI for each study separately.
(DOCX)

Table S2 Risk estimates for SNPs correlated with rs56137030 and combined analyses with rs56137030 for all studies combined.
(DOCX)

Table S3 Functional annotation datasets.
(DOCX)

Table S4 Association between *FTO* SNPs and BMI for SNPs highlighted in previous studies of African Americans.
(DOCX)

Text S1 Description of each study.
(DOC)
