# Supplementary material for: A Systematic Mapping Approach of 16q12.2/FTO and BMI in More Than 20,000 African Americans Narrows in on the Underlying Functional Variation: Results from the Population Architecture using Genomics and Epidemiology (PAGE) Study
Source: PLoS Genet. 2013 Jan 17;9(1):e1003171. doi: 10.1371/journal.pgen.1003171 (PMC3547789; doi:10.1371/journal.pgen.1003171)
Supplement: Figure S1 — Predicted CUX1 binding site at the rs1421085 locus. (DOCX) [file pgen.1003171.s001.docx]

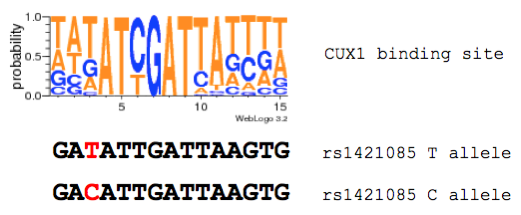


**Figure S1. Predicted CUX1 binding site at the rs1421085 locus.** The TransFac binding site for CUX1, and the *FTO* intron 1 sequence surrounding the rs1421085 locus are shown. When the binding site is aligned with the sequence, rs1421085 is at position 3 of the binding site, where the probability of a T is 0.53 and the probability of a C is 0.06.
