# Supplementary material for: A Systematic Mapping Approach of 16q12.2/FTO and BMI in More Than 20,000 African Americans Narrows in on the Underlying Functional Variation: Results from the Population Architecture using Genomics and Epidemiology (PAGE) Study
Source: PLoS Genet. 2013 Jan 17;9(1):e1003171. doi: 10.1371/journal.pgen.1003171 (PMC3547789; doi:10.1371/journal.pgen.1003171)
Supplement: Table S1 — Association between SNPs in the FTO region and BMI for each study separately. (DOCX) [file pgen.1003171.s002.docx]

| **Table S1: Association between SNPs in the *FTO* region and BMI for each study separately** | | | | | | | |  |  |  |  |  |  |  |  |
| --- | --- | --- | --- | --- | --- | --- | --- | --- | --- | --- | --- | --- | --- | --- | --- |
| **SNP** |  | **Alleles^b^** |  | **CAF^c^** | **% change in BMI per coding allele** | | **nom.p** |  |  |  |  |  |  |  |  |
| **rs#** | **Position^a^** | **Coding** | **Baseline** |  | **Beta estimate** | **95%CI** |  |  |  |  |  |  |  |  |  |
| **ARIC (n=3,297)** | |  |  |  |  |  |  |  |  |  |  |  |  |  |  |
| **Four most significant variants (all in the FTO intron 1 region)** | | | | |  |  |  |  |  |  |  |  |  |  |  |
| rs56137030 | 53825905 | A | G | 0.11 | 1.30 | (-0.17,2.80) | 8.3E-02 |  |  |  |  |  |  |  |  |
| rs62033400 | 53811788 | G | A | 0.11 | 1.08 | (-0.41,2.59) | 1.6E-01 |  |  |  |  |  |  |  |  |
| rs7188250 | 53834607 | C | T | 0.11 | 1.10 | (-0.40,2.62) | 1.5E-01 |  |  |  |  |  |  |  |  |
| rs62033413 | 53830055 | G | C | 0.11 | 1.22 | (-0.29,2.75) | 1.1E-01 |  |  |  |  |  |  |  |  |
|  |  |  |  |  |  |  |  |  |  |  |  |  |  |  |  |
| **Index SNPs of GWAS (variants highlighted in previous studies of EA; all in FTO intron 1 region)** | | | | | | | |  |  |  |  |  |  |  |  |
| rs9939609 | 53820527 | T | A | 0.52 | -0.60 | (-1.49,0.30) | 2.0E-01 |  |  |  |  |  |  |  |  |
| rs8050136 | 53816275 | A | C | 0.44 | 0.52 | (-0.40,1.45) | 2.6E-01 |  |  |  |  |  |  |  |  |
| rs1421085 | 53800954 | G | A | 0.11 | 1.38 | (-0.12,2.90) | 7.1E-02 |  |  |  |  |  |  |  |  |
| rs17817449 | 53813367 | C | A | 0.39 | 0.61 | (-0.31,1.54) | 2.0E-01 |  |  |  |  |  |  |  |  |
| rs1121980 | 53809247 | A | G | 0.47 | 0.37 | (-0.53,1.28) | 4.2E-01 |  |  |  |  |  |  |  |  |
| rs1558902 | 53803574 | T | A | 0.11 | 1.48 | (-0.02,3.00) | 5.3E-02 |  |  |  |  |  |  |  |  |
| rs6499640 | 53769677 | A | G | 0.64 | -0.27 | (-1.20,0.67) | 5.8E-01 |  |  |  |  |  |  |  |  |
| rs9930506 | 53830465 | G | A | 0.21 | 0.17 | (-0.94,1.30) | 7.7E-01 |  |  |  |  |  |  |  |  |
| rs9941349 | 53825488 | A | G | 0.19 | 0.39 | (-0.78,1.58) | 5.1E-01 |  |  |  |  |  |  |  |  |
|  |  |  |  |  |  |  |  |  |  |  |  |  |  |  |  |
| **MEC (n=3,865)** | |  |  |  |  |  |  |  |  |  |  |  |  |  |  |
| **Four most significant variants (all in the FTO intron 1 region)** | | | | |  |  |  |  |  |  |  |  |  |  |  |
| rs56137030 | 53825905 | A | G | 0.13 | 2.15 | (0.92,3.40) | 6.3E-04 |  |  |  |  |  |  |  |  |
| rs62033400 | 53811788 | G | A | 0.12 | 2.20 | (0.95,3.47) | 5.1E-04 |  |  |  |  |  |  |  |  |
| rs7188250 | 53834607 | C | T | 0.12 | 2.22 | (0.97,3.49) | 4.5E-04 |  |  |  |  |  |  |  |  |
| rs62033413 | 53830055 | G | C | 0.13 | 2.39 | (1.13,3.66) | 1.6E-04 |  |  |  |  |  |  |  |  |
| **Index SNPs of GWAS (variants highlighted in previous studies of EA; all in FTO intron 1 region)** | | | | | | | | 53803574 | | T | A | 0.11 | 1.48 | (-0.02,3.00) | 5.3E-02 |
| rs9939609 | 53820527 | T | A | 0.47 | 0.37 | (-0.43,1.18) | 3.7E-01 |  |  |  |  |  |  |  |  |
| rs8050136 | 53816275 | A | C | 0.42 | 1.56 | (0.73,2.40) | 2.2E-04 |  |  |  |  |  |  |  |  |
| rs1421085 | 53800954 | G | A | 0.12 | 1.01 | (-0.24,2.26) | 1.1E-01 |  |  |  |  |  |  |  |  |
| rs17817449 | 53813367 | C | A | 0.38 | 1.46 | (0.63,2.30) | 5.7E-04 |  |  |  |  |  |  |  |  |
| rs1121980 | 53809247 | A | G | 0.46 | 1.21 | (0.40,2.02) | 3.6E-03 |  |  |  |  |  |  |  |  |
| rs1558902 | 53803574 | T | A | 0.14 | 0.54 | (-2.41,3.58) | 7.2E-01 |  |  |  |  |  |  |  |  |
| rs6499640 | 53769677 | A | G | 0.65 | 1.00 | (0.17,1.83) | 1.9E-02 |  |  |  |  |  |  |  |  |
| rs9930506 | 53830465 | G | A | 0.22 | 0.49 | (-0.49,1.48) | 3.3E-01 |  |  |  |  |  |  |  |  |
| rs9941349 | 53825488 | A | G | 0.19 | 0.45 | (-0.59,1.50) | 4.0E-01 |  |  |  |  |  |  |  |  |
| rs9939609 | 53820527 | T | A | 0.47 | 0.37 | (-0.43,1.18) | 3.7E-01 |  |  |  |  |  |  |  |  |
| **GenNet (n=517)** | |  |  |  |  |  |  |  |  |  |  |  |  |  |  |
| **Four most significant variants (all in the FTO intron 1 region)** | | | | |  |  |  |  |  |  |  |  |  |  |  |
| rs56137030 | 53825905 | A | G | 0.10 | 3.64 | (-1.46,9.00) | 1.7E-01 |  |  |  |  |  |  |  |  |
| rs62033400 | 53811788 | G | A | 0.10 | 3.75 | (-1.36,9.12) | 1.5E-01 |  |  |  |  |  |  |  |  |
| rs7188250 | 53834607 | C | T | 0.10 | 3.98 | (-1.15,9.36) | 1.3E-01 |  |  |  |  |  |  |  |  |
| rs62033413 | 53830055 | G | C | 0.10 | 3.96 | (-1.51,9.74) | 1.6E-01 |  |  |  |  |  |  |  |  |
|  |  |  |  |  |  |  |  |  |  |  |  |  |  |  |  |
| **Index SNPs of GWAS (variants highlighted in previous studies of EA; all in FTO intron 1 region)** | | | | | | | |  |  |  |  |  |  |  |  |
| rs9939609 | 53820527 | T | A | 0.50 | -1.66 | (-4.69,1.48) | 3.0E-01 |  |  |  |  |  |  |  |  |
| rs8050136 | 53816275 | A | C | 0.44 | -0.05 | (-3.14,3.13) | 9.7E-01 |  |  |  |  |  |  |  |  |
| rs1421085 | 53800954 | G | A | 0.08 | 4.54 | (-1.06,10.46) | 1.1E-01 |  |  |  |  |  |  |  |  |
| rs17817449 | 53813367 | C | A | 0.59 | 0.71 | (-3.83,2.52) | 6.7E-01 |  |  |  |  |  |  |  |  |
| rs1121980 | 53809247 | A | G | 0.46 | -0.19 | (-3.29,3.01) | 9.1E-01 |  |  |  |  |  |  |  |  |
| rs1558902 | 53803574 | T | A | 0.08 | 4.54 | (-1.06,10.46) | 1.1E-01 |  |  |  |  |  |  |  |  |
| rs6499640 | 53769677 | A | G | 0.62 | -0.12 | (-3.26,3.12) | 9.4E-01 |  |  |  |  |  |  |  |  |
| rs9930506 | 53830465 | G | A | 0.17 | 3.57 | (-0.27,7.57) | 6.9E-02 |  |  |  |  |  |  |  |  |
| rs9941349 | 53825488 | A | G | 0.14 | 3.40 | (-0.81,7.78) | 1.1E-01 |  |  |  |  |  |  |  |  |
| rs9939609 | 53820527 | T | A | 0.50 | -1.66 | (-4.69,1.48) | 3.0E-01 |  |  |  |  |  |  |  |  |
| **HyperGen (n=1,171)** | |  |  |  |  |  |  |  |  |  |  |  |  |  |  |
| **Four most significant variants (all in the FTO intron 1 region)** | | | | |  |  |  |  |  |  |  |  |  |  |  |
| rs56137030 | 53825905 | A | G | 0.10 | 3.30 | (0.23,6.47) | 3.5E-02 |  |  |  |  |  |  |  |  |
| rs62033400 | 53811788 | G | A | 0.09 | 3.45 | (0.36,6.64) | 2.9E-02 |  |  |  |  |  |  |  |  |
| rs7188250 | 53834607 | C | T | 0.09 | 3.54 | (0.47,6.71) | 2.4E-02 |  |  |  |  |  |  |  |  |
| rs62033413 | 53830055 | G | C | 0.09 | 3.68 | (0.55,6.92) | 2.1E-02 |  |  |  |  |  |  |  |  |
| **Index SNPs of GWAS (variants highlighted in previous studies of EA; all in FTO intron 1 region)** | | | | | | | |  |  |  |  |  |  |  |  |
| rs9939609 | 53820527 | T | A | 0.51 | -0.22 | (-2.00,1.60) | 8.1E-01 |  |  |  |  |  |  |  |  |
| rs8050136 | 53816275 | A | C | 0.47 | 0.16 | (-1.63,1.98) | 8.6E-01 |  |  |  |  |  |  |  |  |
| rs1421085 | 53800954 | G | A | 0.09 | 3.43 | (0.31,6.64) | 3.1E-02 |  |  |  |  |  |  |  |  |
| rs17817449 | 53813367 | C | A | 0.58 | 0.47 | (-1.36,2.34) | 6.1E-01 |  |  |  |  |  |  |  |  |
| rs1121980 | 53809247 | A | G | 0.49 | -0.42 | (-2.20,1.39) | 6.4E-01 |  |  |  |  |  |  |  |  |
| rs1558902 | 53803574 | T | A | 0.09 | 3.42 | (0.30,6.63) | 3.2E-02 |  |  |  |  |  |  |  |  |
| rs6499640 | 53769677 | A | G | 0.65 | -1.26 | (-3.06,0.57) | 1.8E-01 |  |  |  |  |  |  |  |  |
| rs9930506 | 53830465 | G | A | 0.19 | 2.44 | (0.14,4.80) | 3.7E-02 |  |  |  |  |  |  |  |  |
| rs9941349 | 53825488 | A | G | 0.16 | 1.63 | (-0.75,4.07) | 1.8E-01 |  |  |  |  |  |  |  |  |
| rs9939609 | 53820527 | T | A | 0.51 | -0.22 | (-2.00,1.60) | 8.1E-01 |  |  |  |  |  |  |  |  |
| **WHI genotyped (=5,312)** | | |  |  |  |  |  |  |  |  |  |  |  |  |  |
| **Four most significant variants (all in the FTO intron 1 region)** | | | | |  |  |  |  |  |  |  |  |  |  |  |
| rs56137030 | 53825905 | A | G | 0.12 | 0.03 | (-1.18,1.25) | 9.7E-01 |  |  |  |  |  |  |  |  |
| rs62033400 | 53811788 | G | A | 0.12 | 0.08 | (-1.15,1.32) | 8.9E-01 |  |  |  |  |  |  |  |  |
| rs7188250 | 53834607 | C | T | 0.12 | -0.02 | (-1.23,1.20) | 9.8E-01 |  |  |  |  |  |  |  |  |
| rs62033413 | 53830055 | G | C | 0.12 | -0.12 | (-1.31,1.08) | 8.4E-01 |  |  |  |  |  |  |  |  |
| **Index SNPs of GWAS (variants highlighted in previous studies of EA; all in FTO intron 1 region)** | | | | | | | |  |  |  |  |  |  |  |  |
| rs9939609 | 53820527 | T | A | 0.53 | 0.16 | (-0.60,0.93) | 6.8E-01 |  |  |  |  |  |  |  |  |
| rs8050136 | 53816275 | A | C | 0.44 | -0.30 | (-1.08,0.49) | 4.5E-01 |  |  |  |  |  |  |  |  |
| rs1421085 | 53800954 | G | A | 0.12 | 0.12 | (-1.09,1.34) | 8.5E-01 |  |  |  |  |  |  |  |  |
| rs17817449 | 53813367 | C | A | 0.40 | -0.21 | (-0.99,0.58) | 6.1E-01 |  |  |  |  |  |  |  |  |
| rs1121980 | 53809247 | A | G | 0.48 | -0.13 | (-0.89,0.64) | 7.4E-01 |  |  |  |  |  |  |  |  |
| rs1558902 | 53803574 | T | A | 0.12 | 0.11 | (-1.1,1.33) | 8.6E-01 |  |  |  |  |  |  |  |  |
| rs6499640 | 53769677 | A | G | 0.65 | -0.19 | (-0.99,0.62) | 6.5E-01 |  |  |  |  |  |  |  |  |
| rs9930506 | 53830465 | G | A | 0.22 | 0.07 | (-0.87,1.02) | 8.9E-01 |  |  |  |  |  |  |  |  |
| rs9941349 | 53825488 | A | G | 0.19 | 0.33 | (-0.67,1.34) | 5.2E-01 |  |  |  |  |  |  |  |  |
| rs9939609 | 53820527 | T | A | 0.53 | 0.16 | (-0.60,0.93) | 6.8E-01 |  |  |  |  |  |  |  |  |
| **WHI imputed (n=6,326)** | | |  |  |  |  |  |  |  |  |  |  |  |  |  |
| **Four most significant variants (all in the FTO intron 1 region)** | | | | |  |  |  |  |  |  |  |  |  |  |  |
| rs56137030 | 53825905 | A | G | 0.13 | 1.45 | (0.38,2.53) | 7.7E-03 |  |  |  |  |  |  |  |  |
| rs62033400 | 53811788 | G | A | 0.13 | 1.45 | (0.38,2.53) | 7.9E-03 |  |  |  |  |  |  |  |  |
| rs7188250 | 53834607 | C | T | 0.13 | 1.46 | (0.39,2.54) | 7.6E-03 |  |  |  |  |  |  |  |  |
| rs62033413 | 53830055 | G | C | 0.13 | 1.37 | (0.30,2.45) | 1.2E-02 |  |  |  |  |  |  |  |  |
| **Index SNPs of GWAS (variants highlighted in previous studies of EA; all in FTO intron 1 region)** | | | | | | | |  |  |  |  |  |  |  |  |
| rs9939609 | 53820527 | T | A | 0.53 | -0.17 | (-0.85,0.52) | 6.2E-01 |  |  |  |  |  |  |  |  |
| rs8050136 | 53816275 | A | C | 0.43 | 0.19 | (-0.49,0.88) | 5.9E-01 |  |  |  |  |  |  |  |  |
| rs1421085 | 53800954 | G | A | 0.12 | 1.42 | (0.33,2.52) | 1.1E-02 |  |  |  |  |  |  |  |  |
| rs17817449 | 53813367 | C | A | 0.40 | 0.08 | (-0.62,0.79) | 8.2E-01 |  |  |  |  |  |  |  |  |
| rs1121980 | 53809247 | A | G | 0.47 | 0.22 | (-0.46,0.91) | 5.3E-01 |  |  |  |  |  |  |  |  |
| rs1558902 | 53803574 | T | A | 0.12 | 1.41 | (0.32,2.51) | 1.1E-02 |  |  |  |  |  |  |  |  |
| rs6499640 | 53769677 | A | G | 0.66 | -0.10 | (-0.82,0.63) | 7.8E-01 |  |  |  |  |  |  |  |  |
| rs9930506 | 53830465 | G | A | 0.22 | 1.15 | (0.30,2.00) | 7.5E-03 |  |  |  |  |  |  |  |  |
| rs9941349 | 53825488 | A | G | 0.20 | 1.09 | (0.20,1.98) | 1.6E-02 |  |  |  |  |  |  |  |  |
| rs9939609 | 53820527 | T | A | 0.53 | -0.17 | (-0.85,0.52) | 6.2E-01 |  |  |  |  |  |  |  |  |
| ^a^SNPposition based on build 37 | | |  |  |  |  |  |  |  |  |  |  |  |  |  |
| ^b^Coding = coding allele, Base= baseline allele (risk estimates provide the log additive effect per copy of the coding allele);  ^c^CAF= coding allele frequency | | | | | | | |  |  |  |  |  |  |  |  |
