# Supplementary material for: A Systematic Mapping Approach of 16q12.2/FTO and BMI in More Than 20,000 African Americans Narrows in on the Underlying Functional Variation: Results from the Population Architecture using Genomics and Epidemiology (PAGE) Study
Source: PLoS Genet. 2013 Jan 17;9(1):e1003171. doi: 10.1371/journal.pgen.1003171 (PMC3547789; doi:10.1371/journal.pgen.1003171)
Supplement: Table S2 — Risk estimates for SNPs correlated with rs56137030 and combined analyses with rs56137030 for all studies combined. (DOCX) [file pgen.1003171.s003.docx]

| **Table S2: Risk estimates for SNPs correlated with rs56137030 and combined analyses with rs56137030 for all studies combined** | | | | | | | | | | | | | |
| --- | --- | --- | --- | --- | --- | --- | --- | --- | --- | --- | --- | --- | --- |
| **SNP** | **Position^a^** | **Allele^b^** |  | **CAF^c^** | **% change in BMI per coding allele^d^** | | **nom.p** | **p.het** | **Correlation with rs56137030** | | **Results for combined analyses with rs56137030^e^** | |  |
|    \| **rs#** \| \| --- \| |  | **Coding** | **Baseline** |  | **beta estimate** | **95%CI** |  |  | **r^2^ in AA** | **r^2^ in EA** | **Nom.p for listed SNP** | **Nom.p for rs56137030** |  |
| **SNPs correlated at r2>0.5 in AA with rs56137030** (ordered by r^2^ with rs56137030 in AA) | | | | | | | | |  |  |  |  |  |
| rs56137030 | 53825905 | A | G | 0.12 | 1.35 | (0.75,1.95) | 8.3E-06 | 1.3E-01 | NA | NA | NA | NA |  |
| chr16:53823727 | 53823727 | C | T | 0.11 | 1.34 | (0.73,1.96) | 1.8E-05 | 6.2E-02 | 0.91 | 0.93 | 8.8E-01 | 2.8E-01 |  |
| rs10468280 | 53827479 | G | A | 0.11 | 1.01 | (0.39,1.62) | 1.4E-03 | 2.5E-01 | 0.91 | 0.98 | 7.2E-01 | 9.2E-01 |  |
| rs11075993 | 53837144 | A | C | 0.12 | 0.98 | (0.36,1.59) | 1.5E-03 | 2.8E-01 | 0.91 | 0.93 | 5.0E-01 | 6.2E-01 |  |
| rs11642841 | 53845487 | A | C | 0.11 | 0.88 | (0.25,1.52) | 5.7E-03 | 2.2E-01 | 0.91 | 0.80 | 8.1E-01 | 2.2E-01 |  |
| rs12149832 | 53842908 | A | G | 0.12 | 0.92 | (0.31,1.54) | 2.5E-03 | 3.8E-01 | 0.91 | 0.93 | 8.3E-01 | 9.3E-01 |  |
| rs17817497 | 53815435 | G | A | 0.11 | 0.99 | (0.37,1.6) | 1.8E-03 | 2.9E-01 | 0.91 | 0.95 | 8.2E-01 | 9.2E-01 |  |
| rs17817712 | 53821125 | G | A | 0.11 | 1.03 | (0.41,1.64) | 1.1E-03 | 2.4E-01 | 0.91 | 0.98 | 5.4E-01 | 9.3E-01 |  |
| rs17817964 | 53828066 | A | G | 0.12 | 1.02 | (0.42,1.61) | 8.6E-04 | 3.5E-01 | 0.91 | 0.98 | 7.9E-01 | 8.3E-01 |  |
| rs3751812 | 53818460 | A | C | 0.11 | 1.02 | (0.40,1.63) | 1.2E-03 | 2.5E-01 | 0.91 | 0.95 | 5.3E-01 | 8.9E-01 |  |
| rs62033405 | 53822387 | T | C | 0.12 | 1.35 | (0.74,1.97) | 1.4E-05 | 6.0E-02 | 0.91 | 0.98 | 6.9E-01 | 4.0E-01 |  |
| rs62033408 | 53827962 | G | A | 0.11 | 1.03 | (0.41,1.64) | 1.1E-03 | 2.6E-01 | 0.91 | 0.98 | 5.4E-01 | 7.0E-01 |  |
| rs7188250 | 53834607 | C | T | 0.12 | 1.34 | (0.74,1.94) | 1.3E-05 | 8.3E-02 | 0.91 | 0.95 | 6.9E-01 | 4.4E-01 |  |
| rs72805611 | 53831354 | A | G | 0.12 | 0.97 | (0.37,1.56) | 1.3E-03 | 3.4E-01 | 0.91 | 0.93 | 8.7E-01 | 8.8E-01 |  |
| rs72805612 | 53834608 | A | G | 0.12 | 1.36 | (0.75,1.98) | 1.4E-05 | 3.9E-02 | 0.91 | 0.95 | 5.4E-01 | 3.0E-01 |  |
| rs72805613 | 53837342 | G | A | 0.12 | 0.89 | (0.30,1.49) | 3.3E-03 | 2.7E-01 | 0.91 | 0.93 | 7.9E-01 | 8.7E-01 |  |
| rs62033413 | 53830055 | G | C | 0.12 | 1.33 | (0.73,1.93) | 1.4E-05 | 4.5E-02 | 0.82 | 0.91 | 5.4E-01 | 3.1E-01 |  |
| rs62033406 | 53824226 | G | A | 0.14 | 0.85 | (0.30,1.41) | 2.5E-03 | 8.6E-02 | 0.75 | 0.95 | 9.1E-01 | 5.5E-03 |  |
| rs11642015 | 53802494 | A | G | 0.11 | 1.09 | (0.47,1.70) | 4.9E-04 | 3.2E-01 | 0.73 | 0.91 | 1.6E-01 | 5.1E-01 |  |
| rs1421085 | 53800954 | G | A | 0.12 | 1.11 | (0.49,1.72) | 3.0E-04 | 2.6E-01 | 0.73 | 0.91 | 8.0E-02 | 5.8E-01 |  |
| rs1558902 | 53803574 | T | A | 0.12 | 1.13 | (0.45,1.80) | 1.0E-03 | 2.4E-01 | 0.73 | 0.91 | 1.2E-01 | 6.0E-01 |  |
| rs55872725 | 53809123 | A | G | 0.11 | 1.09 | (0.47,1.70) | 5.3E-04 | 3.3E-01 | 0.73 | 0.91 | 1.8E-01 | 5.5E-01 |  |
| rs56094641 | 53806453 | G | A | 0.12 | 1.12 | (0.50,1.73) | 2.8E-04 | 2.7E-01 | 0.73 | 0.91 | 6.4E-02 | 4.6E-01 |  |
| rs62033400 | 53811788 | G | A | 0.12 | 1.35 | (0.75,1.95) | 1.1E-05 | 1.3E-01 | 0.73 | 0.95 | 9.0E-01 | 6.2E-01 |  |
| rs62048402 | 53803223 | A | G | 0.12 | 1.13 | (0.51,1.74) | 2.4E-04 | 2.6E-01 | 0.73 | 0.91 | 6.5E-02 | 5.1E-01 |  |
| rs11649091 | 53845169 | G | T | 0.16 | 1.10 | (0.54,1.65) | 9.1E-05 | 1.4E-01 | 0.69 | 0.87 | 7.3E-01 | 3.1E-02 |  |
| rs9922619 | 53831771 | A | C | 0.19 | 0.69 | (0.20,1.19) | 5.7E-03 | 4.5E-01 | 0.55 | 0.87 | 4.7E-01 | 2.3E-01 |  |
| rs9931494 | 53827179 | G | C | 0.19 | 0.94 | (0.45,1.44) | 1.6E-04 | 3.4E-01 | 0.55 | 0.91 | 5.3E-01 | 1.6E-02 |  |
|  |  |  |  |  |  |  |  |  |  |  |  |  |  |
| **SNPs correlated at r2>0.2 to <0.5 in AA with rs56137030** (ordered by r^2^ with rs56137030 in AA) | | | | | | | | |  |  |  |  |  |
| rs11646715 | 53824007 | A | G | 0.19 | 0.84 | (0.33,1.36) | 1.1E-03 | 2.2E-02 | 0.48 | 0.64 | 8.6E-01 | 2.2E-03 |  |
| chr16:53817318 | 53817318 | A | G | 0.06 | 1.23 | (0.36,2.10) | 5.7E-03 | 1.1E-01 | 0.47 | 0.18 | 7.4E-01 | 6.5E-04 |  |
| rs11647020 | 53823990 | T | C | 0.20 | 0.73 | (0.24,1.23) | 3.5E-03 | 1.3E-02 | 0.42 | 0.65 | 6.4E-01 | 7.2E-04 |  |
| rs28432761 | 53823878 | C | T | 0.19 | 0.83 | (0.34,1.33) | 9.7E-04 | 2.6E-02 | 0.42 | 0.65 | 9.1E-01 | 2.4E-03 |  |
| rs9941349 | 53825488 | A | G | 0.19 | 0.69 | (0.20,1.19) | 5.4E-03 | 5.7E-01 | 0.42 | 0.91 | 5.4E-01 | 2.5E-01 |  |
| rs9922708 | 53831146 | A | G | 0.21 | 0.64 | (0.13,1.16) | 1.6E-02 | 1.1E-01 | 0.40 | 0.89 | 5.5E-01 | 6.7E-01 |  |
| rs9930506 | 53830465 | G | A | 0.22 | 0.66 | (0.19,1.14) | 5.5E-03 | 1.6E-01 | 0.38 | 0.87 | 3.0E-01 | 1.3E-01 |  |
| rs9933040 | 53830867 | T | A | 0.22 | 0.65 | (0.18,1.13) | 6.0E-03 | 1.6E-01 | 0.38 | 0.87 | 3.6E-01 | 1.3E-01 |  |
| rs9927317 | 53820996 | G | C | 0.26 | 0.85 | (0.42,1.29) | 1.5E-04 | 3.1E-02 | 0.34 | 0.98 | 1.4E-01 | 6.4E-03 |  |
| rs72803664 | 53784911 | G | A | 0.89 | 0.13 | (-0.57,0.84) | 7.1E-01 | 1.5E-01 | 0.32 | 0.11 | 5.5E-02 | 1.8E-06 |  |
| rs28567725 | 53826028 | C | T | 0.22 | 0.87 | (0.4,1.35) | 2.4E-04 | 1.9E-01 | 0.32 | 0.91 | 4.2E-01 | 7.8E-03 |  |
|  |  |  |  |  |  |  |  |  |  |  |  |  |  |
| **Index SNPs of GWAS** (variants highlighted in previous studies of EA; all in *FTO* intron 1 region) | | | | | | | | | |  |  |  |  |
| rs9939609 | 53820527 | T | A | 0.52 | 0.04 | (-0.33,0.41) | 8.2E-01 | 5.5E-01 | 0.14 | 0.95 | 4.5E-01 | 2.9E-02 |  |
| rs8050136 | 53816275 | A | C | 0.44 | 0.42 | (0.03,0.82) | 3.2E-02 | 4.9E-02 | 0.15 | 0.95 | 6.5E-01 | 5.5E-02 |  |
| rs1421085 | see above |  |  |  |  |  |  |  |  |  |  |  |  |
| rs17817449 | 53813367 | C | A | 0.39 | 0.37 | (-0.02,0.77) | 5.9E-02 | 5.6E-02 | 0.14 | 0.95 | 9.9E-01 | 6.8E-02 |  |
| rs1121980 | 53809247 | A | G | 0.47 | 0.35 | (-0.02,0.73) | 7.2E-02 | 2.4E-01 | 0.09 | 0.85 | 8.3E-01 | 4.3E-02 |  |
| rs1558902 | see above |  |  |  |  |  |  |  |  |  |  |  |  |
| rs6499640 | 53769677 | A | G | 0.65 | 0.05 | (-0.34,0.44) | 8.1E-01 | 1.6E-01 | 0.00 | 0.12 | 9.1E-01 | 8.0E-03 |  |
|    \| rs9930506 \| \| --- \| | see above |  |  |  |  |  |  |  |  |  |  |  |  |
| rs9941349 | see above |  |  |  |  |  |  |  |  |  |  |  |  |
| ^a^SNPposition based on build 37 | | |  |  |  |  |  |  |  |  |  |  |  |
| ^b^Coding = coding allele, Base= baseline allele (risk estimates provide the log additive effect per copy of the coding allele);  ^c^CAF= coding allele frequency | | | | | | | | | | | |  |  |
| ^d^Association analysis that only includes the listed SNP | | | | | |  |  |  |  |  |  |  |  |
| ^e^Association analysis with rs56137030 and a listed SNP in the same model | | | | | | | |  |  |  |  |  |  |
