# Supplementary material for: A Systematic Mapping Approach of 16q12.2/FTO and BMI in More Than 20,000 African Americans Narrows in on the Underlying Functional Variation: Results from the Population Architecture using Genomics and Epidemiology (PAGE) Study
Source: PLoS Genet. 2013 Jan 17;9(1):e1003171. doi: 10.1371/journal.pgen.1003171 (PMC3547789; doi:10.1371/journal.pgen.1003171)
Supplement: Table S3 — Functional annotation datasets. (DOCX) [file pgen.1003171.s004.docx]

| **Table S3. Functional annotation datasets** | | |  |
| --- | --- | --- | --- |
| **Dataset** | **Genomic class** | **Description** | **Data source/program** |
| 1 | Non-synonymous coding | Exonic positions wherein the variant would cause an amino acid replacement | dbSNP version 131 |
| 2 | Promoter | 1kb regions upstream of annotated transcription start sites | RefSeq |
| 3 | Promoter TFBS | Transcription factor binding sites (TFBS) predicted in promoter regions | PWM-scan^a^ |
| 4 | Non-coding RNA | All types of experimentally supported non-coding RNA, including microRNAs | RNAdb 2.0 & miRBase 17.0 |
| 5 | MicroRNA target site | Computationally predicted microRNA target sites within 3’ UTRs | TargetScanS 5.2 |
| 6 | Enhancer element | Experimentally supported enhancer elements in any tissue | VISTA Enhancer Browser |
| 7 | Candidate non-promoter regulatory element | Open chromatin loci in at least one human cell type, as assessed by DNase I hypersensitivity (DHS) mapping | UCSC Table Browser: Duke and UW DNase I HS data from > 50 cell types |
| 8 | Candidate non-promoter regulatory element TFBS | Transcription factor binding sites (TFBS) predicted in candidate non-promoter regulatory elements | PWM-scan^a^ |
| 9 | Predicted transcriptional regulatory module | Computationally predicted *cis*-regulatory modules | PReMod database |
| 10 | Insulator elements | CTCF binding sites assessed by ChIP-chip technology | UCSC Table Browser: ORegAnno |
| 11 | MCS elements | Most Conserved Sequences across 17 vertebrates | UCSC Table Browser: 17-way most conserved |
| ^a^PWM-scan was applied using positional weight matrices (PWMs) from the Transfac database | | | |
|  |  |  |  |
