# Supplementary material for: A Systematic Mapping Approach of 16q12.2/FTO and BMI in More Than 20,000 African Americans Narrows in on the Underlying Functional Variation: Results from the Population Architecture using Genomics and Epidemiology (PAGE) Study
Source: PLoS Genet. 2013 Jan 17;9(1):e1003171. doi: 10.1371/journal.pgen.1003171 (PMC3547789; doi:10.1371/journal.pgen.1003171)
Supplement: Table S4 — Association between FTO SNPs and BMI for SNPs highlighted in previous studies of African Americans. (DOCX) [file pgen.1003171.s005.docx]

| **Table S4. Association between *FTO* SNPs and BMI for SNPs highlighted in previous studies of African Americans** | | | | | | | | | |
| --- | --- | --- | --- | --- | --- | --- | --- | --- | --- |
| **SNP** |  | **Alleles^b^** |  | **CAF^c^** | **% change in BMI per coding allele** | | **p-value** | **Rsq^d^** | **p.het** |
| **rs#** | **Position^a^** | **Coding** | **Baseline** |  | **Beta estimate** | **95%CI** |  |  |  |
|  |  |  |  |  |  |  |  |  |  |
| **within FTO intron 1 region** | | |  |  |  |  |  |  |  |
| rs3751812 | 53818460 | A | C | 0.11 | 1.02 | (0.40,1.63) | 1.2E-03 | 1.00^f^ | 2.5E-01 |
| rs8057044 | 53812614 | A | G | 0.71 | 0.27 | (-0.14,0.68) | 2.1E-01 | 1.00^f^ | 9.7E-02 |
| rs16945088 | 53812524 | A | G | 0.71 | 0.16 | (-0.25,0.57) | 4.5E-01 | 1.00^f^ | 1.6E-01 |
| rs1108102 | 53789508 | T | A | 0.86 | 0.09 | (-0.46,0.64) | 7.4E-01 | 1.00^f^ | 9.0E-01 |
| rs8044769 | 53839135 | G | A | 0.75 | 0.33 | (-0.10,0.76) | 1.3E-01 | 1.00^f^ | 5.0E-01 |
| rs7204609 | 53833605 | A | G | 0.66 | 0.16 | (-0.25,0.57) | 4.3E-01 | 1.00^f^ | 6.8E-02 |
| rs9941349 | 53825488 | A | G | 0.19 | 0.69 | (0.20,1.19) | 5.4E-03 | 1.00^f^ | 5.7E-02 |
|  |  |  |  |  |  |  |  |  |  |
| **outside of FTO intron 1 region** | | |  |  |  |  |  |  |  |
| rs9932411 | 54005163 | A | G | 0.33 | 0.12 | (-0.29,0.53) | 5.5E-01 | 1.00^f^ | 8.3E-01 |
| rs7191513 | 53990523 | A | G | 0.54 | 0.03 | (-0.36,0.42) | 8.7E-01 | 1.00^f^ | 7.3E-01 |
| ^a^SNPposition based on build 37 | | |  |  |  |  |  |  |  |
| ^b^Coding = coding allele, Base= baseline allele (risk estimates provide the log additive effect per copy of the coding allele)  ^c^CAF= coding allele frequency | | | | | | | | | |
| ^d^Rsq = measurement of imputation accuracy, ranging from 0 (low) to 1 (high)  ^f^SNP was on directly genotyped on the metabochip | | | | | | | | |  |
