# Supplementary material for: A Systematic Mapping Approach of 16q12.2/FTO and BMI in More Than 20,000 African Americans Narrows in on the Underlying Functional Variation: Results from the Population Architecture using Genomics and Epidemiology (PAGE) Study
Source: PLoS Genet. 2013 Jan 17;9(1):e1003171. doi: 10.1371/journal.pgen.1003171 (PMC3547789; doi:10.1371/journal.pgen.1003171)
Supplement: Text S1 — Description of each study. (DOC) [file pgen.1003171.s006.doc]

**Notes S1 “A systematic mapping approach of 16q12.2/*FTO* and BMI in over 20,000 African Americans narrows in on the underlying functional variation: results from the Population Architecture using Genomics and Epidemiology (PAGE) Study”**

**Peters et al.**

**Descriptions of studies**

The Population Architecture using Genomics and Epidemiology (PAGE) study is a National Human Genome Research Institute funded effort examining the epidemiologic architecture of common genetic variants that have been reproducibly associated with human diseases and traits (https://www.pagestudy.org). The PAGE study consists of a coordinating center and four consortia, each of which with access to large, diverse population-based studies including the Atherosclerosis Risk in Communities study, the Coronary Artery Risk Disease in Young Adults study, the Cardiovascular Health Study, the Hispanic Community Health Study/Study of Latinos, three National Health and Nutrition Examination Surveys, the Multiethnic Cohort, the Strong Heart Study, and the Strong Heart Family Study and the Women’s Health Initiative. For specific analyses as for this paper PAGE reached out to additional studies, such as GenNet and Hypertension Genetic Epidemiology Network to expand the sample size.

**Atherosclerosis Risk in Communities Study (ARIC)**. This cohort is part of Causal Variants Across the Life Course (CALiCo) a consortium of well characterized population based studies and a central genotyping and resequencing core laboratory. This collaborative network includes six of the most informative and demographically diverse population-based studies extant, contributing approximately 58,000 men and women from the main ethnic and racial groups in the U.S., ranging in age from childhood to old adulthood. ARIC is a multi-center prospective investigation of atherosclerotic disease in a predominantly bi-racial population conducted in four U.S. communities, involving both cohort and community surveillance components. European American and African American (AA) men and women aged 45-64 years at baseline were recruited from 4 communities: Forsyth County, North Carolina; Jackson, Mississippi; suburban areas of Minneapolis, Minnesota; and Washington County, Maryland. A total of 15,792 individuals participated in the baseline examination in 1987-1989, with follow-up examinations in approximate 3-year intervals, during 1990-1992, 1993-1995, and 1996-1998. Weight and height were measured. All AA subjects in ARIC were genotyping on the Metabochip.

**Hypertension Genetic Epidemiology Network (HyerGEN)** is part of the Family Blood Pressure Program funded by the National Heart Lung and Blood Institute and was designed to study the genetics of hypertension and related conditions. Participants were recruited from multiply-affected hypertensive sibships ascertained through population-based cohorts or from the community-at-large. The study was later extended to include siblings and offspring of the original sibpair. Probands were identified by the onset of hypertension before age 60 and the presence of at least one additional hypertensive sibling who was willing to participate. Participants with type 1 diabetes or advanced renal disease (defined as serum creatinine level >2 mg/dL) were excluded from the original study since these two conditions can cause secondary hypertension and the goal of HyperGEN was to identify novel essential hypertension loci. Recruitment, cross-sectional clinical measurement, and DNA isolation were completed in 2003. Two of four centers (AL, NC) recruited 1,264 AAs, while three centers (NC, MN, and UT) recruited Caucasians . All AA were genotyped on the Metabochip and included in this analysis.

**GenNet** recruited African-American (n=1101) and European-American participants (n=1497) at two field-centers between 1995 and 2003, based on a hypertensive proband. Non-Hispanic white subjects were recruited from Tecumseh, Michigan, and African-American subjects were recruited from Maywood, Illinois. Probands were defined as individuals aged 18–50 years with blood pressures in the upper 20th to 25th percentile of the age/gender-specific blood pressure distribution. Once the proband was identified, an attempt was made to enroll all siblings and parents of the proband, irrespective of their blood pressure or hypertension treatment status . All AA hypertensive probands were genotyped on the Metabochip and included in the analysis.

**The Multiethnic Cohort (MEC)** is a population-based prospective cohort study of over 215,000 men and women in Hawaii and California aged 45-75 at baseline (1993-1996) and primarily of five ancestries . The MEC was funded by the National Cancer Institute in 1993 to examine lifestyle risk factors and genetic susceptibility to cancer. All eligible cohort members completed baseline and follow-up questionnaires. AA subjects were selected for Metabochip genotyping based on availability of biomarker for cardiovascular risk factors or from a pool of controls for a study of type 2 diabetes.

**The Women’s Health Initiative (WHI)** is a prospective cohort study investigating post-menopausal women’s health in the U.S . WHI was funded by the National Institutes of Health and the National Heart, Lung, and Blood Institute to study strategies to prevent heart disease, breast cancer, colon cancer, and osteoporotic fractures in women aged 50-79. WHI consists of two parts: randomized clinical trials of hormone therapy, dietary modification, and calcium/Vitamin D supplementation, and an observational cohort study. All AA subjects who provided informed consent to submit their genotype data to dbGaP where either directly genotyped on the Metabochip or had genome-wide data on the Affymetrix 6.0 array available to impute Metabochip SNPs (details see Methods).

**References**

1 Matise, T.C., Ambite, J.L., Buyske, S., Carlson, C.S., Cole, S.A., Crawford, D.C., Haiman, C.A., Heiss, G., Kooperberg, C., Marchand, L.L. *et al.* (2011) The Next PAGE in understanding complex traits: design for the analysis of Population Architecture Using Genetics and Epidemiology (PAGE) Study. *Am J Epidemiol*, **174**, 849-859.

2 (1989) The Atherosclerosis Risk in Communities (ARIC) Study: design and objectives. The ARIC investigators. *Am J Epidemiol*, **129**, 687-702.

3 Williams, R.R., Rao, D.C., Ellison, R.C., Arnett, D.K., Heiss, G., Oberman, A., Eckfeldt, J.H., Leppert, M.F., Province, M.A., Mockrin, S.C. *et al.* (2000) NHLBI family blood pressure program: methodology and recruitment in the HyperGEN network. Hypertension genetic epidemiology network. *Ann Epidemiol*, **10**, 389-400.

4 (2002) Multi-center genetic study of hypertension: The Family Blood Pressure Program (FBPP). *Hypertension*, **39**, 3-9.

5 Kolonel, L.N., Henderson, B.E., Hankin, J.H., Nomura, A.M., Wilkens, L.R., Pike, M.C., Stram, D.O., Monroe, K.R., Earle, M.E. and Nagamine, F.S. (2000) A multiethnic cohort in Hawaii and Los Angeles: baseline characteristics. *Am J Epidemiol*, **151**, 346-357.

6 (1998) Design of the Women's Health Initiative clinical trial and observational study. The Women's Health Initiative Study Group. *Control Clin Trials*, **19**, 61-109.
